# Caffeine Augments Anesthesia Neurotoxicity in the Fetal Macaque Brain

**DOI:** 10.1038/s41598-018-23560-7

**Published:** 2018-03-28

**Authors:** Kevin K. Noguchi, Stephen A. Johnson, Francesca M. Manzella, Kobe L. Masuoka, Sasha L. Williams, Lauren D. Martin, Gregory A. Dissen, Chrysanthy Ikonomidou, Katie J. Schenning, John W. Olney, Ansgar M. Brambrink

**Affiliations:** 1 Department of Psychiatry, Washington University in St Louis, St. Louis, MO 63130 USA; 2 Mayo Clinic, Department of Neurology, Rochester, Minnesota United States; 3 Department of Anesthesiology, Neuroscience Program, University of Colorado Anschutz Medical Campus, Aurora, CO 80045 USA; 4 Department of Psychology, University of Missouri, St Louis, USA; 5 Division of Comparative Medicine, Oregon Health & Science University, Beaverton, OR 97006 USA; 6 Division of Neuroscience, Oregon Health & Science University, Beaverton, OR 97006 USA; 7 Department of Neurology, University of Wisconsin-Madison, Madison, WI 53705 USA; 8 Department of Anesthesiology & Perioperative Medicine, Oregon Health & Science University, Portland, OR 97239 USA; 9 Department of Anesthesiology, Columbia University Irving Medical Center/New York Presbyterian Hospital, New York, NY 10032 USA

## Abstract

Caffeine is the most frequently used medication in premature infants. It is the respiratory stimulant of choice for apnea associated with prematurity and has been called the silver bullet in neonatology because of many proven benefits and few known risks. Research has revealed that sedative/anesthetic drugs trigger apoptotic death of neurons and oligodendrocytes in developing mammalian brains. Here we evaluated the influence of caffeine on the neurotoxicity of anesthesia in developing nonhuman primate brains. Fetal macaques (n = 7–8/group), at a neurodevelopmental age comparable to premature human infants, were exposed *in utero* for 5 hours to no drug (control), isoflurane, or isoflurane + caffeine and examined for evidence of apoptosis. Isoflurane exposure increased apoptosis 3.3 fold for neurons and 3.4 fold for oligodendrocytes compared to control brains. Isoflurane + caffeine caused neuronal apoptosis to increase 8.0 fold compared to control levels but did not augment oligoapoptosis. Neuronal death was particularly pronounced in the basal ganglia and cerebellum. Higher blood levels of caffeine within the range considered therapeutic and safe for human infants correlated with increased neuroapoptosis. Caffeine markedly augments neurotoxicity of isoflurane in the fetal macaque brain and challenges the assumption that caffeine is safe for premature infants.

## Introduction

Neurodevelopmental deficits are prominent among survivors of premature birth, particularly those born with a very low birth weight (<1500 grams; VLBW). Fifty percent of VLBW infants demonstrate impaired academic achievement and behavioral disorders^1^ while brain imaging has revealed increased CSF volume (indicative of loss of brain mass) in addition to prominent reductions in cerebral cortical grey matter and deep nuclear grey matter^2^. Interestingly, birth prior to 32 weeks gestation is associated with a significant alteration in grey matter structure and adverse neurodevelopmental outcomes at 1 year of age. Pathophysiology of abnormal brain volumes and structure in VLBW infants is likely multifactorial and in most cases the etiologic factors remain unknown. Notably, new concerns have emerged that iatrogenic contributions may alter the normal developmental trajectory of the brain.

Sedative, anesthetic, and anti-epileptic^3–5^ drugs (SADs) frequently used in neonatal medicine can trigger widespread apoptosis of neurons and oligodendrocytes in the developing brain of several animal species (including non-human primates) and produce long-term neurodevelopmental impairment^6–10^. Whether anesthetics produce similar toxicity in humans is still heavily debated and is supported by some clinical studies^11–17^ but not others^18–20^. However, in December 2016 the Federal Drug Administration required eleven commonly used SADs to include labels warning drug exposure exceeding 3 hours may harm the developing brain. This change surprised many clinicians, remains quite controversial, and will change medical care for the millions of children exposed to these drugs each year^21^. As a result, there is a great need to study what factors influence SAD-induced neurotoxicity.

VLBW premature infants exposed to anesthesia plus surgery have shown stunted brains and deep nuclear grey matter volumes in addition to increased incidence of neurodevelopmental impairment^22,23^. VLBW infants are often treated with SADs intermittently or continuously over days or weeks while simultaneously receiving caffeine (CAF) to prevent apnea of prematurity. Because surgical anesthesia can increase apnea, CAF is administered by many practitioners during the perioperative period^24^. CAF has recently been referred to as the ‘silver bullet of neonatology’^25–27^ because of its safety and putative therapeutic benefits, including a decrease in prevalence of bronchopulmonary dysplasia^28^ and cerebral palsy^29^. This has led to CAF being the most commonly used drug in the neonatal intensive care unit (NICU) for premature infants^30^.

This practice is concerning in view of recent reports^31,32^ demonstrating that administration of CAF to rats causes neuroapoptosis in the developing brain. Prompted by these findings, our group administered CAF to infant mice in combination with several SADs and found that CAF markedly potentiated the neuroapoptotic response to each of these agents^33,34^.

We hypothesized that CAF potentiates apoptosis in the developing primate brain and undertook the present study to determine the impact of concomitant CAF treatment on the documented neurotoxic effects of general anesthetics^4^. Non-human primate fetuses at an age comparable to that of premature human infants were exposed for 5 hours to the anesthetic isoflurane (ISO), ISO + CAF, or to no drug (control), and after a 3-hour recovery period their brains were histopathologically examined.

## Results

### Tolerance of general anesthesia and caffeine administration

**Figure 1:**
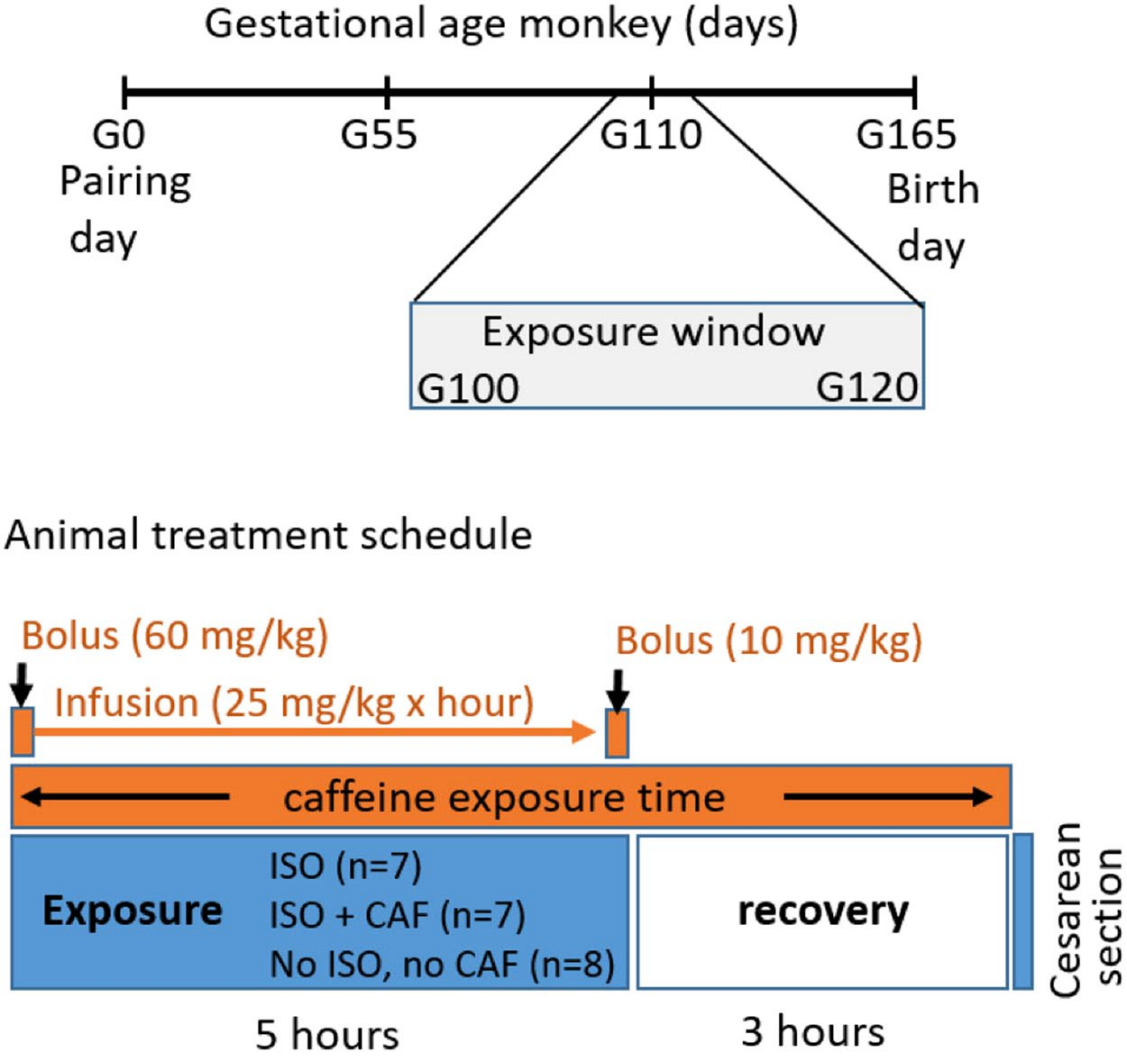
Animal exposure window and treatment schedule used to expose fetal monkeys to isoflurane and caffeine. (**A**) Fetal monkeys were exposed to one of three treatments *in utero* between G100 and G120 days gestational age. (**B**) Dams were exposed to either ISO, ISO + CAF, or no exposed controls (no ISO no CAF) for 5 hours and allowed to recover for 3 hours before a cesarean section under anesthesia to collect the fetal brains. Caffeine was administered as an initial bolus followed by continuous infusion during isoflurane exposure and a final bolus before the recovery period. Caffeine blood levels were measured at the time of the cesarean section. (**C**) The doses of caffeine administrated over the 5-hour period and caffeine blood levels are indicated in the table.

Pregnant animals were either exposed to ISO, ISO + CAF, or saline as a control (Fig. 1). Animals tolerated induction and maintenance of general anesthesia and their vital signs remained within physiological limits without specific intervention as described previously^4,5^. Intravenous caffeine administration was generally well tolerated by the dams randomized to this treatment condition. We observed transient tachypnea associated with each caffeine bolus, and higher respiration rates as the experiment continued. We also observed transient trends towards higher heart rate and blood pressures, which were resolved by the end of the 3-hour observation period. Animals that received high-dose caffeine developed mild muscular rigidity that resolved before induction of anesthesia for cesarean section. Animals that received high-dose caffeine treatment also developed mild nystagmus early in the experiment, while with low-dose caffeine this phenomenon was only observed in a few animals towards the end of the experiment (hours 4–5). Trans-abdominal ultrasound did not reveal any abnormalities in fetal heart rate or activity. While recovering from ISO anesthesia, CAF exposed dams appeared more alert and active as compared to animals that did not receive CAF treatment. No lingering effects were noted during routine veterinarian evaluation 24 hours following the experiment. Isoflurane requirements in caffeine treated animals were not higher than in animals that did not receive caffeine. Similarly, caffeine did not hasten recovery from ISO anesthesia or influence time to extubation of the trachea or transfer from the operating room table to the recovery cage.

### Pattern of cell death and cell types affected

In fluorescent double staining experiments, we found many dying cellular profiles that stained positive for AC3 (apoptosis marker) and those in the gray matter co-labeled for AC3 and NeuN, confirming their neuronal identity. Dying cellular profiles in the white matter co-labeled for AC3 and myelin basic protein (MBP), which identified these cells as oligodendrocytes, because oligodendrocytes are the only cell type in the brain that contains MBP. None of the AC3-positive cells in either the gray or white matter co-labeled for AC3 and any other marker, including GFAP (marker for astrocytes) or Iba1 (marker for microglia and macrophages). Therefore, we concluded that ISO alone and ISO + CAF cause the same type of cytopathology involving apoptotic cell death of neurons in the gray matter and oligodendrocytes in the white matter.

**Figure 2:**
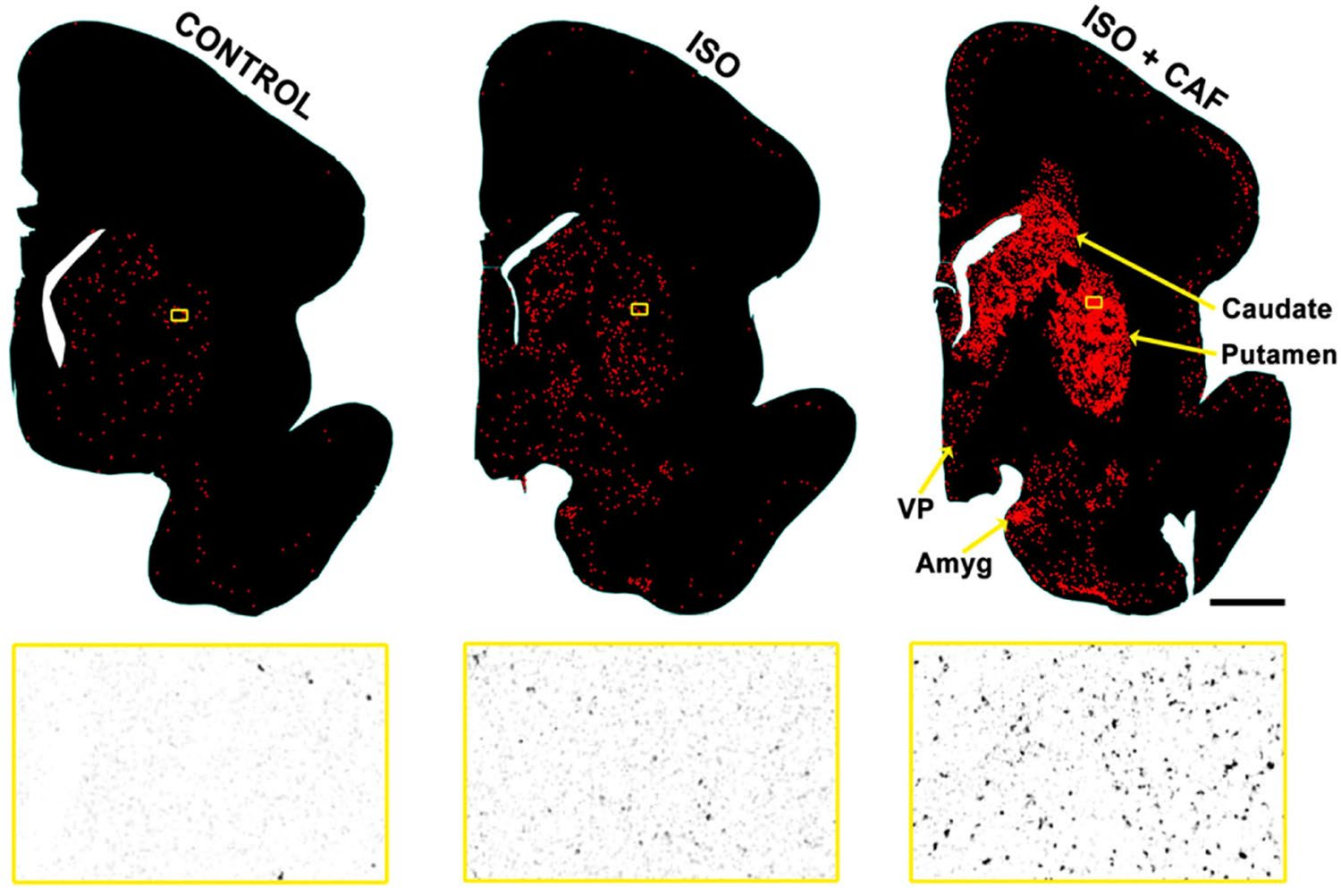
Caffeine (CAF) augmentation of isoflurane (ISO) apoptosis at basal ganglia level in the G100 fetus. Each density plot is a computer image showing the outline of a tissue section cut at the level of the basal ganglia (caudate/putamen) and nearby gray matter zones (ventral pallidum - VP; amygdala - Amyg) which collectively have been referred to in the recent human neuroimaging literature as the deep nuclear gray matter. Each red dot marks the location of an apoptotic neuron (activated caspase-3 positive cell) and large amounts of toxicity can be seen in the basal ganglia (the main nuclear mass of the deep nuclear grey matter). Each density plot contains an outlined rectangle (yellow) that is magnified in an image below. Compared to the control group, the deep nuclear grey matter has an increased number of apoptotic neurons following ISO treatment with a far greater increase following ISO + CAF. Scale Bar = 3 mm.

We also determined that the pattern of cell death was very similar at G100 and G120. At both ages a striking feature of the pathological reaction to ISO was a particularly high density of dying neurons in a region that has been referred to in recent human neuroimaging (MRI) literature as the deep nuclear gray matter. This region consists of the basal ganglia (caudate/putamen) and nearby gray matter zones (nucleus accumbens, ventral pallidum, amygdala, and anterior thalamus). Neurons in the deep nuclear grey matter showed a robust apoptotic response to ISO and this response was markedly augmented by exposure to CAF + ISO (Fig. 2). In prior studies pertaining to G120 non-human primate fetuses, various anesthetic drugs (ketamine, propofol, isoflurane) and alcohol have shown a predilection for deleting large numbers of neurons from the deep nuclear grey matter region^4,5,35^. These same apoptogenic agents preferentially delete neurons from other brain regions at other developmental ages^3,5,35^. Both G100 and G120 ISO deleted large numbers of oligodendrocytes from the developing brain and were distributed diffusely throughout the white matter wherever active myelination was occurring.

**Figure 3:**
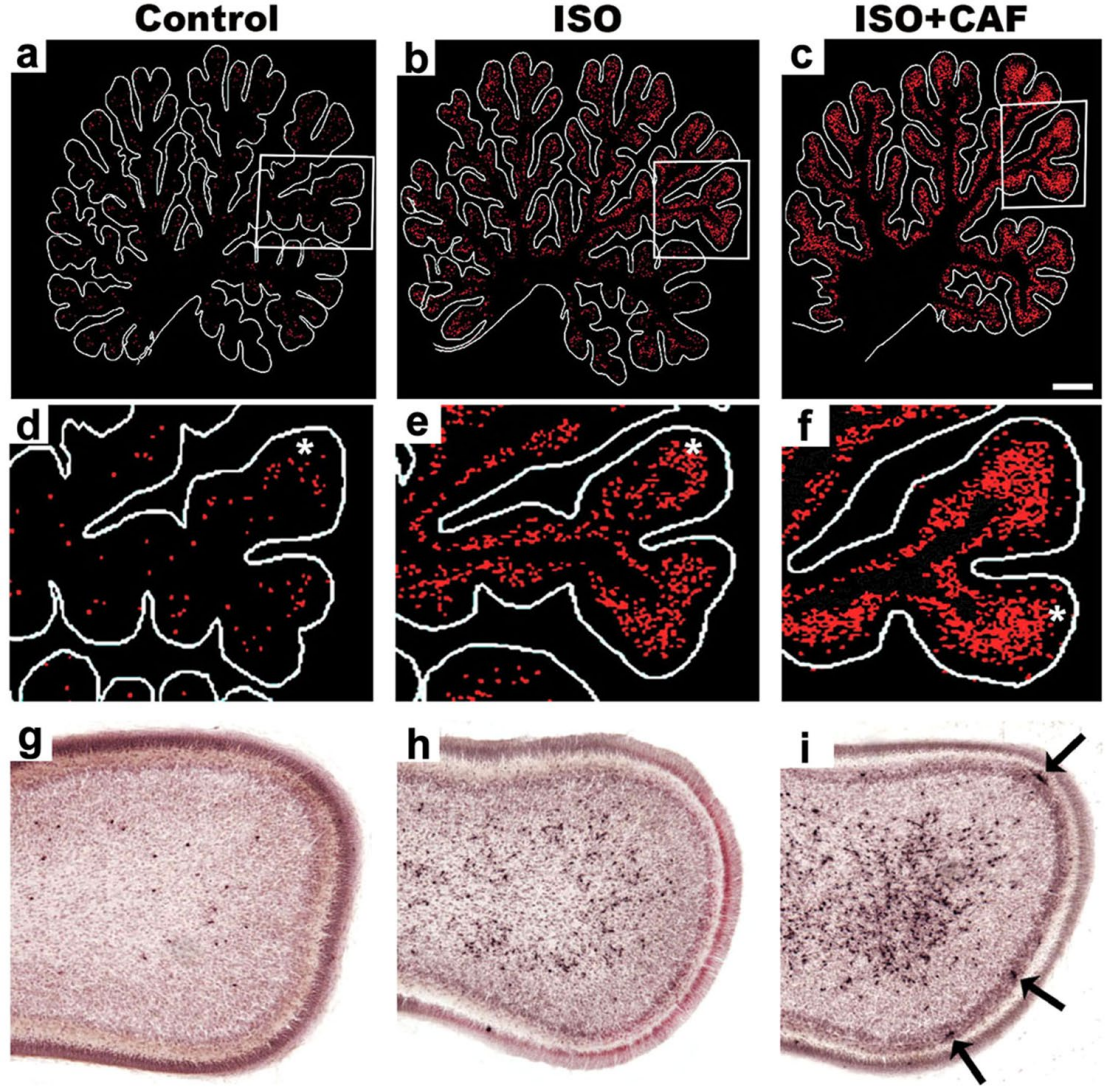
Caffeine (CAF) augmentation of isoflurane (ISO) neuroapoptosis in the cerebellum. Panels A–C are computer plots of sagittal sections cut through the cerebellum 2 mm lateral to the mid-sagittal plane from brains exposed to no drug (Control), isoflurane (ISO), or ISO + CAF. Panels D–F are magnified views from the boxed regions in (**A**–**C**). Panels G–I are histological sections stained with antibodies to activated caspase 3 (apoptosis marker) to show the histological appearance of the dying neurons that are represented as red dots on the computer plots. The asterisks in (**D**,**E** and **F)** indicate the lobule that is depicted histologically in (**G**,**H** and **I**). In the infant mouse brain and the fetal monkey brain this population of cerebellar neurons is exquisitely sensitive to the neurotoxic action of alcohol (37–38) and anesthetic drugs (11). These cells are undergoing migration toward the Purkinje cell layer where they are thought to modulate Purkinje cell function. In panel I, note that there are some dying Purkinje cells (arrows) that are not seen in G or H, signifying that CAF + ISO is more toxic to Purkinje cells than ISO alone. Scale Bar = 1 mm.

Another striking feature of the pathological reaction to ISO, which appeared to be more extreme in CAF + ISO brains, was vulnerability of a specific population of neurons in the cerebellum (Fig. 3). These neurons have also been noted previously to be vulnerable to the apoptogenic action of multiple anesthetic drugs^4,5,35^ and alcohol^36^ in the non-human primate brain at G120, and our present observations extend the vulnerable period for these neurons back to G100.

### Quantitative histopathology

**Figure 4:**
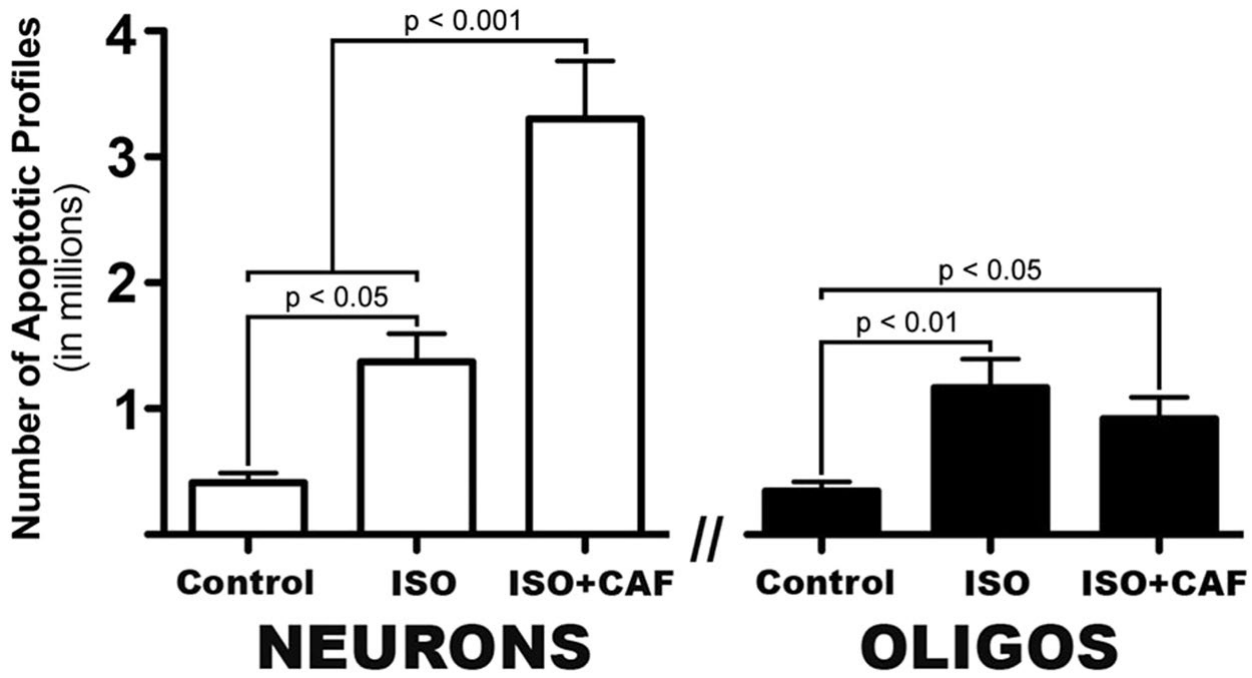
Total number of apoptotic neurons or oligodendrocytes per brain following exposure to no drug (Control), isoflurane (ISO), or ISO + caffeine (ISO + CAF). A one-way ANOVA on the number of apoptotic neurons and oligodendrocytes (oligos) revealed a statistically significant difference between groups (F[2,19] = 26.80, p < 0.0001 and F[2,19] = 7.037, p < 0.01 respectively). ISO alone significantly increased the amount of neuronal and oligo apoptosis when compared to controls. However, CAF + ISO further increased apoptosis compared to ISO for only neurons.

Quantitative evaluation of AC3-stained sections revealed that the group exposed to ISO alone (n = 7) had a mean (±SEM) number of apoptotic neuronal profiles 3.3 times higher than the mean for the control group (n = 8) (1.37 × 10^6^ ± 0.22 × 10^6^ vs 0.41 × 10^6^ ± 0.07 × 10^6^; P < 0.05), whereas the mean number of apoptotic neurons for the ISO + CAF group (n = 7) (3.30 × 10^6^ ± 0.46 × 10^6^) was 8.0 times higher than the control mean (P < 0.001). The mean for the ISO + CAF group was significantly higher than the mean for the ISO group (P < 0.001) (Fig. 4).

Counts pertaining to apoptotic oligodendrocytes in the white matter revealed that the group exposed to ISO alone (n = 7) had a mean (±SEM) number of apoptotic oligodendrocyte profiles 3.4 times higher than the mean for the control group (n = 8) (1.17 × 10^6^ ± 0.23 × 10^6^ vs 0.35 × 10^6^ ± 0.07 × 10^6^; P < 0.01), whereas the mean for the ISO + CAF group (n = 7) (0.92 × 10^6^ ± 0.17 × 10^6^) was not significantly different (P = 0.7) from the mean for the ISO group, signifying that CAF did not augment the apoptogenic action of ISO on oligodendrocytes (Fig. 4).

### Caffeine blood levels

**Figure 5:**
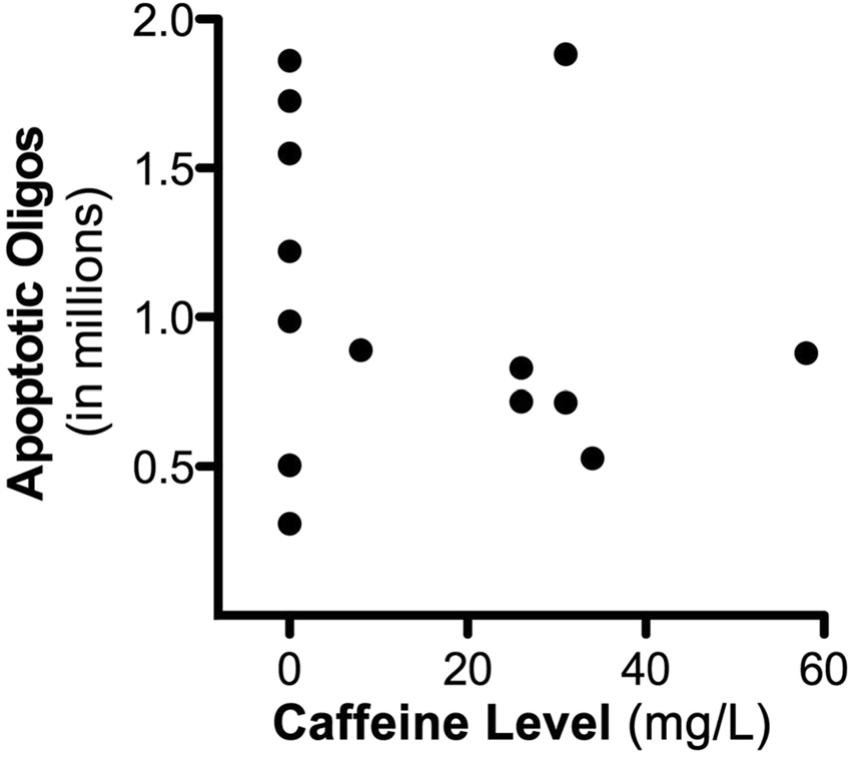
Comparison of caffeine blood levels and oligodendrocyte apoptosis during isoflurane anesthesia. Plot of the total number of apoptotic oligodendrocytes (oligos) per brain compared to caffeine blood levels during isoflurane anesthesia. A Pearson’s r coefficient was run and revealed no statistically significant positive linear correlation indicating that increasing caffeine levels was not correlated with heightened neuroapoptosis (r = −0.2072, p > 0.05).

**Figure 6:**
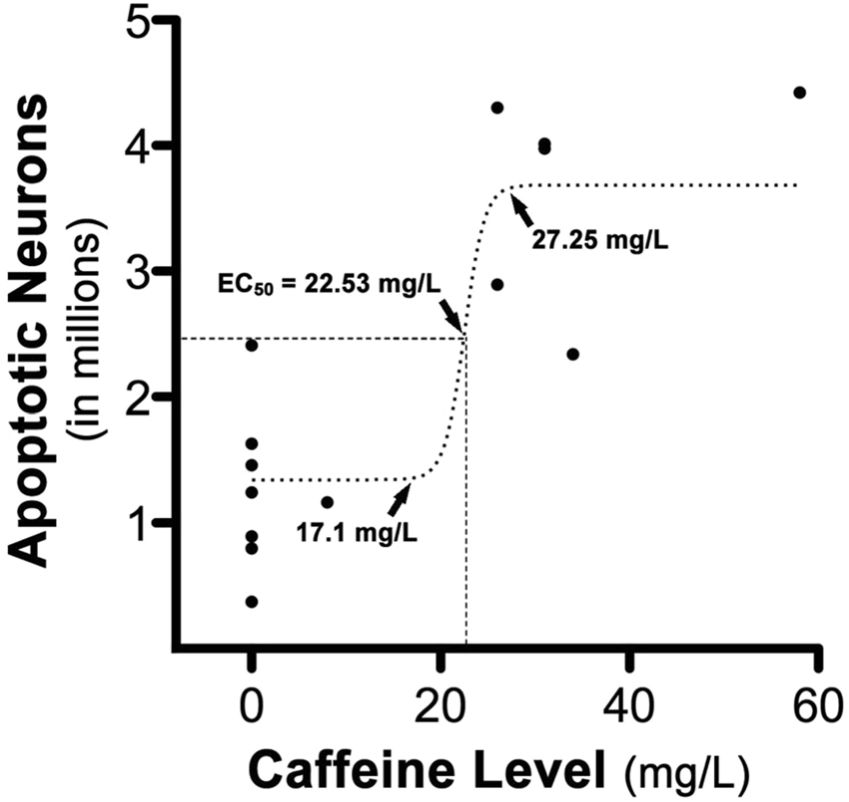
Comparison of caffeine blood levels and neuroapoptosis during isoflurane anesthesia. Plot of the total number of apoptotic neurons per brain compared to caffeine blood levels during isoflurane anesthesia. A subsequent Pearson’s r correlation coefficient revealed a statistically significant positive linear correlation indicating that increasing caffeine levels were correlated with heightened neuroapoptosis (r = 0.8438, p < 0.0001). To examine this dose-response relationship in more detail, CAF levels and neuronal apoptotic counts were further analyzed by producing a best-fit four-parameter logistics equation resulting in a sigmoidal (dotted) curve with an R^2^ of 0.7471 and LogEC_50_ of 22.53 (dashed lines). Analysis of this curve can be used to estimate the amount of apoptosis that would be expected based on the dose of CAF. For instance, as CAF levels increased from 17.1 to 27.25 mg/L (arrows at top and bottom of curve), the estimated neuronal apoptosis would increase from 1.359 to 3.661 million (almost 3-fold) respectively.

There were 7 fetuses that received CAF in addition to ISO. For the dams of each of these fetuses we calculated the mean CAF blood level averaged over the 5-hour period during which ISO exposure of the fetus occurred (the measured CAF plasma levels were 8, 26, 26, 31, 31, 34 and 58 mg/L). We also measured the fetal blood level at the time of cesarian section and found that it was essentially identical to the 5-hour average for the dam. The blood levels for all dams are plotted against the fetal apoptosis scores for oligodendrocytes (Fig. 5) and neurons (Fig. 6). Caffeine levels of zero correspond to dams that received ISO without caffeine (i.e. the ISO group). A Pearson’s r correlation between CAF levels and oligoapoptosis was not statistically significant (r = −0.2072, p = 0.4773) indicating a lack of dose-response relationship between these parameters. As a result, this data was not examined further. We next examined the relationship between CAF levels and neuronal apoptosis by running a Pearson’s r correlation. Results revealed a statistically significant positive linear correlation indicating that increasing CAF levels correlated with heightened apoptosis (r = 0.8438, p < 0.0001). To analyze this data in more detail, a best-fit four-parameter logistics equation was generated resulting in a sigmoidal curve with: an EC_50_ of 22.53 mg/L, an R^2^ of 0.7471, a Bottom of 1.346, a Top of 3.687, and a Hillslope of 0.4124. Analysis of this curve indicated that apoptotic counts increased dramatically as CAF levels increased above 17.1 mg/L. For example, as CAF levels increased from 17.1 to 27.25 mg/L apoptotic counts increased 2.7 fold (Fig. 6).

## Discussion

CAF is considered a safe and beneficial drug for use in neonatal medicine^24–27^, but recent evidence from infant rodent studies raised concerns that it can trigger apoptosis of neurons in the developing brain, as can SADs^31,32^. When administered with SADs, CAF consistently augments their apoptogenic action^33,34^. Our findings re-establish that the anesthetic ISO induces apoptosis affecting both neurons and oligodendrocytes in the fetal monkey brain at a developmental age comparable to that of a premature human infant (for comparison, we previously reported isoflurane increased the number of apoptotic profiles [neurons + oligodendroctes] 4.1-fold in the fetal macaque similar to the 3.4-fold increase in the current study). However, we additionally show CAF significantly augments neuronal apoptosis over 8-fold but has no significant effect on oligodendrocytes. These findings are of concern in that premature infants are commonly exposed to CAF to prevent apnea, while also being exposed to SADs for procedural sedation and/or surgical interventions.

We administered CAF according to dosing schedules calculated to produce blood levels in a range considered safe and beneficial for very preterm human infants. Several research groups have argued that routine monitoring of blood levels of CAF in very preterm infants may be unnecessary^25–27^. CAF citrate can be administered at a bolus of 80 mg/kg with daily maintenance dose of 20 mg/kg for weeks without causing adverse effects or any apparent long-term neurobehavioral deficits at the 1-year follow-up. CAF blood levels associated with this dosing schedule range from 19–80 mg/L, and a much lower dosing schedule (20 mg/kg bolus followed by 5 mg/kg daily maintenance) produces blood levels in the range of 5–25 mg/L^27^. The CAF blood levels in the 7 non-human primate subjects in the present study ranged from 8–58 mg/L (median = 31 mg/L) and, therefore, were within the range considered safe and beneficial for very preterm human infants. Analysis of the relationship between CAF blood levels and apoptotic brain injury revealed that ISO-induced brain injury increases sharply as CAF blood levels rise above 17 mg/L. This suggests that there may not be a wide safety margin for exposing very preterm infants to CAF in combination with other apoptogenic drugs.

Isoflurane, sevoflurane, and desflurane, are commonly used in pediatric medicine as surgical anesthetics. Because they can cause apnea, CAF is sometimes used during the peri-anesthesia period to bolster respiratory effort^24^. It was recently reported that very preterm infants exposed to surgery have an increased risk for neurodevelopmental impairment^22,23^, and MRI evidence for structural brain injury^22^, which is particularly prominent in a region referred to as deep nuclear gray matter. Omizzolo *et al*.^37^ have defined the deep nuclear grey matter as a region consisting of the basal ganglia and surrounding gray matter zones, and have reported that MRI-documented structural pathology in the deep nuclear grey matter region is a frequent finding in very preterm infants, especially those who later display poor neurodevelopmental outcomes. Our current findings help to establish that the deep nuclear grey matter region in the nonhuman primate brain is at peak vulnerability to the apoptogenic action of alcohol and anesthetic drugs during a period extending from G100 to G120, which is comparable in neurodevelopmental age to the period that human very preterm infants spend in the NICU. Other evidence lending potential significance to these findings is: 1) Human fetuses exposed *in utero* to alcohol frequently have MRI-documented loss of neuronal mass in the basal ganglia (deep nuclear grey matter) region^38,39^, coupled with poor neurodevelopmental outcomes (i.e., fetal alcohol spectrum disorder - FASD); 2) Exposure of human fetuses *in utero* to anti-epileptic drugs during the third trimester is associated with reduced brain volume localized specifically to the basal ganglia (deep nuclear grey matter) region^40^, and *in utero* exposure to anti-epileptic drugs is associated with a measurable deficit in IQ and/or impairment in verbal communication skills^41,42^. Thus, exposure to these pharmacologically similar drugs that can trigger extensive apoptosis in the developing animal brain^10^, have been identified in the human literature as agents associated with increased risk for long term neurodevelopmental impairment and pronounced structural pathology in the deep nuclear grey matter region of the developing human brain.

For procedural sedation very preterm infants are commonly exposed to a SAD in the benzodiazepine class, either intermittently or continuously for prolonged periods (days, weeks, months) while they are often also exposed continuously to CAF. Our previous research demonstrates that prolonged SAD exposures produce higher amounts of neurotoxicity in both the non-human primate^3,43^ and rodent^44^. These findings together with our newest observations suggest that intermittent or continuous SAD exposures when combined with CAF may be particularly dangerous to the developing infant brain. It has been demonstrated in infant mice that a 4-hour exposure to a sub-anesthetic dose of midazolam^45^ or diazepam^33^ induces a significant neurotoxic reaction, and when CAF is administered together with either drug, the neurotoxic action is significantly augmented^33,34^.

It has been demonstrated that all anesthetic drugs currently used in pediatric medicine rapidly trigger neuroapoptosis in the developing animal brain^10^. When CAF was administered together with an apoptogenic agent (alcohol, phencyclidine, ketamine, diazepam, isoflurane), it significantly augmented neuroapoptosis^33,34^. Therefore, it is highly unlikely that the synergistic toxicity shown in the present study between CAF and ISO is due to a specific property of ISO. It is more likely potentiation is related to caffeine’s ability to non-specifically antagonize adenosine receptors (A_1_, A_2a_, A_2b_, and A_3_). Interestingly, since stimulation of A_2a_ increases glutamate release, caffeine may act as a glutamate antagonist^46^. We have previously found glutamate antagonists (such as alcohol, MK-801, and phencyclidine) potently increase apoptosis in the developing brain that is augmented when combined with GABA agonists^47^. It is important to point out that the augmenting effects of CAF are not limited to agents that independently cause neuroapoptosis in the developing brain. Black and colleagues^32^ have reported that CAF triggers cell death in the developing rat brain while morphine does not. However, when CAF and morphine were administered together the apoptogenic action of CAF was significantly augmented^31^. Given that opioid analgesics are frequently administered to very preterm infants receiving CAF, with or without a SAD, it will be important to systematically examine all drugs that very preterm infants receive during their first weeks of life, either individually or in combination, in order to determine the potential for contribution to the poor neurodevelopmental outcomes that is reported for very preterm infants^1,37^.

While it seems like a contradiction that CAF could promote apoptosis but be protective against cerebral palsy and neurodevelopmental impairment, these are not mutually exclusive possibilities. CAF is used in pediatric medicine over a wide range of doses and a wide range of blood levels (6–80 mg/L)^26,27^. In the study by Schmidt *et al*.^29^ describing CAF as being protective against cerebral palsy and neurodevelopmental impairment, it is noteworthy in that: 1) The CAF dose used would produce blood levels between 5–25 mg/L^27^ which in our experiments showed little effect on ISO-induced apoptosis (Fig. 6) 2) No information was given regarding the amount of SAD exposure for either the CAF-exposed or control infants; 3) The benefit pertaining to cerebral palsy and neurodevelopmental impairment is apparently ephemeral - for both conditions the benefit was considered weakly significant at 18–21 months of age, but was absent in the same cohort at 5 yrs^48^. Therefore, it is possible that CAF at moderate to high doses, together with prolonged exposure to SADs, could induce brain injury and contribute to poor neurobehavioral outcomes, while CAF at low doses with relatively little co-exposure to SADs could be beneficial due to prevention of apnea, while at the same time not damaging to the brain even when administered together with SADs. Consistent with this, a recent study found that high dose caffeine was associated with neurobehavioral deficits and increased cerebellar injury when compared to a low dose^49^.

In summary, a large body of evidence identifies drugs used in the routine care of very preterm infants, including CAF and SADs, as apoptogenic agents that can cause long term neurodevelopmental impairment. Here we report that one such SAD induces robust neuroapoptosis in the fetal monkey brain at a neurodevelopmental age comparable to that of a human very preterm infant, and CAF markedly augments that reaction. It has been assumed that CAF is safe for very preterm infants over a wide range of doses^24–27^, but this assumption does not take into consideration that clinically CAF is often administered in the context of other drug treatments including sedatives or anesthetics. Our findings call attention to the need for a more detailed safety evaluation of CAF in neonatal intensive care medicine and during the perioperative period.

## Methods

### Animals and Experimental Procedures

The study protocol and all related procedures were conducted with the approval of the Institutional Animal Care and Use Committees of the Oregon National Primate Research Center at Oregon Health and Science University and Washington University Medical School, in addition to being in full accordance with the Public Health Service Policy on Humane Care and Use of Laboratory Animals. The methods used to provide general anesthesia for the study animals were similar to those previously reported^35,50^ for anesthesia exposure of nonhuman primate fetuses, and were in keeping with standards for human pediatric anesthesia currently endorsed by the American Society of Anesthesiologists and similar professional societies.

The subjects were time-mated pregnant rhesus macaques; the females were paired with males for 4 days beginning on approximately day 10 of the menstrual cycle. Gestation length was counted from the first day of pairing (day 0); for full description of the breeding method see reference^35^.

At gestational age 100–120 days (G100–120; n = 22; full term for rhesus macaques = 165 days), the pregnant dams were exposed for 5 hours to ISO (n = 7), ISO + CAF (n = 7) or no anesthesia (Controls; n = 8) (Fig. 1). Gestational ages between G100 and G120 were chosen because these two ages bracket a developmental stage in the rhesus macaque that is comparable to that of a human VLBW infant (~28 weeks gestation). Five animals in the ISO group and four in the control group were G120 animals used in a previous paper^4^ in which stored sections were restained and counted for the current study. Due to the higher ethical standards when working with non-human primates, it would be difficult to justify sacrificing nine different fetal macaques simply to replicate our previous finding^4^ that ISO is neurotoxic compared to controls.

### General Anesthesia

ISO anesthesia was administered as previously described (Fig. 1)^4^. Briefly, following IV catheter placement, dams received a propofol bolus (2 mg/kg IV), were placed on standard *American Society of Anesthesiologists*-monitoring, and were tracheally intubated for airway protection and to allow precise application of ISO in addition to mechanical ventilation (direct laryngoscopy using a endotracheal tube with an internal diameter of 4.0–5.0 millimeters [Mallinckrodt, Hazelwood, MO]; veterinary ventilator, Hallowell Engineering & Manufacturing Corporation [Pittsfield, MA]; end-tidal 1.0–1.5 Vol%; Capnomac [Datex Ohmeda, Madison, WI]). ISO was titrated according to a pre-defined clinical endpoint that represents an intermediate surgical plane of anesthesia (deep nail-bed stimulation at hand and foot [mosquito-clamp pinch] does not result in motor response and only a mild sympathetic response evidenced by 10% or less increase in heart rate or blood pressure; monitored every 30 min)^4^. This type of response to the standardized deep nail-bed stimulation is typical for a level of anesthesia that would allow skin incision and the initiation of a moderate surgical intervention. Anesthetic management was aimed at maintaining homeostasis. Extended physiologic monitoring included end-tidal gas measurements, electrocardiogram, direct blood pressure, peripheral oxygen saturation and esophageal temperature as well as regular blood gas and metabolic status evaluations. Intravenous fluids and glucose were provided (as clinically indicated) and body temperature was maintained at physiologic levels using a warming blanket (Eden Prairie, MN) and forced warm air (Bear Hugger, Arizant Healthcare Inc.). Fetal heart rate was evaluated every hour using ultrasonography.

### Caffeine Administration

CAF is administered to preterm infants over a wide range of doses because it is believed to be beneficial and safe over a wide range of blood levels (6–80 mg/L)^27^. CAF therapy is typically initiated by a bolus dose intended to produce a desired blood CAF level and then is supplemented every 24 hours with a maintenance dose to achieve a steady state level in the desired range. Therapy is often initiated by a 20 mg/kg bolus dose of CAF citrate followed by a daily maintenance dose of 5 mg/kg, which results in serum CAF levels up to 25 mg/L, but a higher dose is currently recommended for the peri-extubation period to assist in weaning the premature infant off the ventilator without incurring episodes of apnea. Similarly, caffeine is frequently administrated to preterm infants when they have to undergo anesthesia for a surgical or diagnostic procedure up to 56 weeks postconceptual age. For this purpose, CAF citrate is administered as a bolus dose at 80 mg/kg followed by a daily maintenance dose of 20 mg/kg, which results in serum CAF levels from 19 to 80 mg/L^27^. The blood levels reported from the clinical laboratory and in the literature do not reflect peak blood levels because the blood sample is often drawn during the “trough” period immediately prior to administering a maintenance booster. In preparation for the present study, we performed a pilot study (data not shown) aimed at establishing a dosing regimen that we predicted would produce steady-state CAF blood levels within the clinically relevant range of 6 to 80 mg/L with the aim of most of the values being in the middle of that range. Informed by this pilot study, when CAF citrate was administered, it was given as an initial bolus dose with propofol ranging from 20 to 60 mg/kg and supplanted with a constant infusion of CAF in the range of 5 to 25 mg/kg/h during the course of anesthesia, followed by a final bolus of 0 to 10 mg/kg as illustrated in Fig. 1.

Recovery, post-anesthesia observation, cesarean section, and brain preparation were performed as previously reported^4^. In brief, at 5 hours from time zero (i.e. beginning of anesthesia), ISO was discontinued; the dams were extubated (within approximately 5–10 min of the end of ISO administration) and returned to an ICU cage to allow close post-procedural monitoring for the remainder of the 3-hour observation period. At 8 hours after time zero, general anesthesia was induced again according to the above strategy, followed by immediate cesarean section under aseptic conditions and following state-of-the-art standard operating procedures established at Oregon National Primate Research Center (skin incision to delivery <5 min). Samples from umbilical venous and arterial blood were used to determine fetal and placental well-being, as well as terminal CAF blood levels in the ISO + CAF group. After brief morphologic measurements (body weight; dimensions of hand, foot, biparietal diameter, crown-to-rump; <1 min), the fetus received IV pentobarbital (umbilical vein) to induce a deep barbiturate coma and allow *in-vivo* transcardial perfusion-fixation according to NIH guidelines in order to prepare the brain for histopathological analysis.

After delivery of the fetus, the dam was managed according to Oregon National Primate Research Center standard operating procedures (balanced anesthesia including local anesthetics and opioid analgesics until completion of operative procedure; appropriate postoperative analgesia; return to home cage).

Dams randomized to the control group (no anesthesia; n = 8) were handled in the same manner as described above for the ISO-treated animals, however instead of intubation and induction of general anesthesia, these animals received IV saline and were returned to their cage with access to water *ad libitum* for the remainder of the experimental period (Fig. 1). At 8 hours after time zero, the fetus was removed via cesarean section under general anesthesia. Fetal *in-vivo* brain perfusion was conducted as described above, and the dam was managed according to the above paradigm^4^.

### Histopathology methods

In previous studies^3–5,35,50^, we applied a battery of immunohistochemical and other histological procedures to characterize the cell death process. In the present study, we applied the same methods to determine whether the brains exposed to ISO + CAF have pathological changes that are qualitatively the same or different from those induced by ISO alone. In addition, since the prior studies pertained to infants or fetuses at age G120, we wanted to determine whether the pathological changes in G100 brains are different from those in G120 brains. Methods employed include immunohistochemical staining with antibodies to activated caspase 3 (AC3, a reliable marker for apoptotic cell death), immunofluorescent double labeling with antibodies to AC3 and NeuN (marker for neurons), myelin basic protein (marker for oligodendrocytes), glial fibrillary acidic protein (GFAP, marker for astrocytes), or Iba1 (marker for microglia and macrophages). Further details regarding staining protocols and source of reagents are given in recently published articles^3–5,35,50^.

### Quantification of apoptosis

The stereological quantitative analysis of cellular apoptosis was conducted by an investigator blinded to the treatment conditions using serial sections cut at 2 mm intervals across the entire brain, and stained with AC3 to mark apoptotic profiles. Sections were quantified at 10x magnification using a computer-assisted Microbrightfield Stereo-Investigator system to estimate the number of AC3-positive (apoptotic) profiles. It was possible to distinguish apoptotic neurons from apoptotic oligodendrocytes because, as we have illustrated previously^3–5,35,50^, the AC3 stain displays the full cell body and processes of both cell types, and they are morphologically dissimilar. Moreover, the dying oligodendrocytes are confined almost exclusively to white matter and the dying neurons are confined exclusively to gray matter.

### Statistical analysis

Data are presented as mean ± standard error of the mean (SEM). Statistically significant one-way ANOVAs were followed by the Newman Keuls post-hoc test for multiple comparisons. For comparisons of caffeine levels and apoptosis a Pearson’s r correlation coefficient was used to test linear correlations and a best-fit sigmoidal curve was generated using a four-parameter logistics equation. All statistics were performed using GraphPad Prism software, version 4.0a (GraphPad Software, Inc., La Jolla, CA).

### Data Availability Statement

The datasets generated during and/or analysed during the current study are available from the corresponding author on reasonable request.
